# Analysis of using the tongue deviation angle as a warning sign of a stroke

**DOI:** 10.1186/1475-925X-11-53

**Published:** 2012-08-21

**Authors:** Ching-Chuan Wei, Shu-Wen Huang, Sheng-Lin Hsu, Hsing-Chung Chen, Jong-Shin Chen, Hsinying Liang

**Affiliations:** 1 Department of Information and Communication Engineering, Chaoyang University of Technology, Taichung, Taiwan; 2 Department of Health, Executive Yuan, Graduate Institute of Informatics, Chaoyang University of Technology and Taichung Hospital, Taichung, Taiwan; 3 Department of Computer Science and Information Engineering, Asia University, Taichung, Taiwan; 4, 168, Jifeng E. Rd., Wufeng District, Taichung, 41349, Taiwan

**Keywords:** Tongue, Deviation angle, Stroke, Transient ischemic attack, Quantification

## Abstract

**Background:**

The symptom of tongue deviation is observed in a stroke or transient ischemic attack. Nevertheless, there is much room for the interpretation of the tongue deviation test. The crucial factor is the lack of an effective quantification method of tongue deviation. If we can quantify the features of the tongue deviation and scientifically verify the relationship between the deviation angle and a stroke, the information provided by the tongue will be helpful in recognizing a warning of a stroke.

**Methods:**

In this study, a quantification method of the tongue deviation angle was proposed for the first time to characterize stroke patients. We captured the tongue images of stroke patients (15 males and 10 females, ranging between 55 and 82 years of age); transient ischemic attack (TIA) patients (16 males and 9 females, ranging between 53 and 79 years of age); and normal subjects (14 males and 11 females, ranging between 52 and 80 years of age) to analyze whether the method is effective. In addition, we used the receiver operating characteristic curve (ROC) for the sensitivity analysis, and determined the threshold value of the tongue deviation angle for the warning sign of a stroke.

**Results:**

The means and standard deviations of the tongue deviation angles of the stroke, TIA, and normal groups were: 6.9 ± 3.1, 4.9 ± 2.1 and 1.4 ± 0.8 degrees, respectively. Analyzed by the unpaired Student’s *t*-test, the *p-*value between the stroke group and the TIA group was 0.015 (>0.01), indicating no significant difference in the tongue deviation angle. The *p-*values between the stroke group and the normal group, as well as between the TIA group and the normal group were both less than 0.01. These results show the significant differences in the tongue deviation angle between the patient groups (stroke and TIA patients) and the normal group. These results also imply that the tongue deviation angle can effectively identify the patient group (stroke and TIA patients) and the normal group. With respect to the visual examination, 40% and 32% of stroke patients, 24% and 16% of TIA patients, and 4% and 0% of normal subjects were found to have tongue deviations when physicians “A” and “B” examined them. The variation showed the essentiality of the quantification method in a clinical setting. In the receiver operating characteristic curve (ROC), the Area Under Curve (AUC, = 0.96) indicates good discrimination. The tongue deviation angle more than the optimum threshold value (= 3.2°) predicts a risk of stroke.

**Conclusions:**

In summary, we developed an effective quantification method to characterize the tongue deviation angle, and we confirmed the feasibility of recognizing the tongue deviation angle as an early warning sign of an impending stroke.

## Introduction

Stroke, a cerebral vascular incident, is mainly caused by abnormal blood vessels in the brain. According to the statistical results of the World Health Organization (WHO), stroke remains the worldwide second leading cause of death. It is estimated that one in five stroke survivors will have the chance of a second stroke within five years. Thus, it has a high recurrence rate, and recurrence can bring about disability and dementia, often leading to a heavy burden for an individual household, a community, and ultimately society in general. This reminds us how important it is to prevent, recognize and monitor the stroke subject.

A stroke, often occurring suddenly, happens for two main reasons. Firstly, a hemorrhagic stroke results from a weakened vessel that ruptures and bleeds into the surrounding brain. About 20% of strokes are hemorrhagic. Secondly, and more commonly, an ischemic stroke occurs when an artery in the brain becomes blocked. About 80% of strokes are ischemic strokes. If a major artery to the brain is blocked, part of the brain tissue can die from lack of oxygen carried in the blood. When brain tissue dies, the effects on the body will depend on which body functions that part of the brain controls. Some of the effects on the body are quite well known and are commonly recognized as the result of a stroke. A stroke will produce changes in the body and affect various functions, including the sensory function, action function, language ability, the swallowing function, etc. For instance, paralysis of the right side of the body (right-sided stroke) will be caused by damage to the left half of the brain. In most patients, inability to speak will also be due to damage to the left side of the brain, because the left side of the brain controls speech.

A “mini-stroke,” also called a “transient ischemic attack” (TIA), causes a reversible neurologic deficit. The damaged area can be so small that the blood supply to other parts of the brain can compensate, and a full recovery takes place. Dizziness and giddiness, blurred vision, and unsteadiness are the typical symptoms of a TIA. These symptoms resolve themselves in less than 24 hours. People often disregard the mini-stroke, failing to recognize that the TIA may be a warning sign that a more severe stroke may take place [1]. It is not uncommon for those who experience but disregard the TIA to have a stroke several days later. Therefore, recognizing the symptoms of a TIA and recognizing the TIA as a warning may make it possible to prevent a stroke.

The tongue in mammals has important motor and sensory functions. When the motor cortex in the brain is damaged, the hypoglossal nerve, which is a pure motor nerve innervating the muscles of the tongue, will be defective. Therefore, the tongue will have a tendency to turn away from the midline when extended or protruded, and it will deviate toward the side of the lesion. This is called tongue deviation [2-5]. Hence, the symptom of tongue deviation is observed in a stroke or TIA [5-8]. For thousands of years, the deviation of the tongue has also been recognized as a symptom of what is called a “wind stroke” in traditional Chinese medicine [9-11]. The symptom of tongue deviation in stroke patients has been observed from ancient to modern times. Many people may recognize the more common symptoms of a stroke, such as slurred speech or paralysis of one side of the body; however, fewer are familiar with tongue deviation.

Nevertheless, there is much room for the interpretation of the tongue deviation test. How crooked is crooked? How far to one side does the tongue have to be regarded as a clear sign of a stroke having occurred? There are too many variables with tongue deviation. The crucial factor is the lack of an effective quantification method of tongue deviation. If we can quantify the features of the tongue deviation and scientifically verify the relationship between the deviation angle and a stroke, the information provided by the tongue will be helpful in recognizing a warning sign of a stroke. In this study, we developed a simple and effective method to quantify the deviation angle of the crooked tongue, and we conducted the experiments to verify the feasibility of using the tongue deviation angle as the warning sign of a stroke. Finally, we used the receiver operating characteristic curve (ROC) for the sensitivity analysis, and determined the threshold value of the tongue deviation angle for the warning sign of a stroke.

## Methods

### Tongue image acquisition and edge segmentation

**Figure 1:**
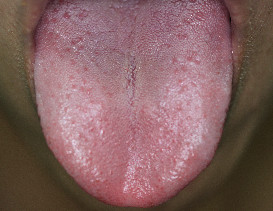
The captured tongue image.

**Figure 2:**
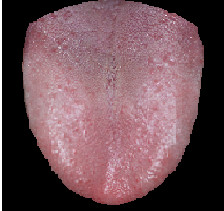
The pure tongue image through edge segmentation.

We built a brace rack to support the chin in order to fix the tongue position. Subjects were asked to make the tongue protrude. Then, we took a picture including the subject’s tongue using a digital camera and a circular light source, which uniformly distributes the light on the tongue, as shown in Figure 1. Next, we adopted the threshold method using Otsu’s thresholding algorithm and filtering process for edge segmentation because they can achieve an easy, fast, and effective segmentation result with the tongue image [12]. Thus, we were able to remove skin, tooth, lip, background, etc., to obtain the pure tongue image, as shown in Figure 2[12-15]. In the following steps, we will start to quantify the angle of tongue deviation. 

### Quantifying the angle of tongue deviation

#### Locating the root point

**Figure 3:**
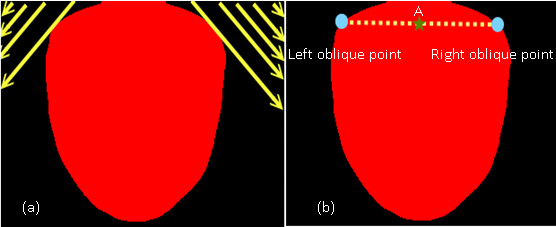
(a) The searching order of the left and right obliques; (b) The star marks the root point of the tongue.

The first step is to locate the root point of the tongue, which is defined as the center point at the bottom of the tongue. Before locating the root point, the left side and right side points of the tongue bottom should be found. These two points were searched for using the oblique angle method, described in the following and demonstrated in Figure 3(a).

1. The searching order of the left oblique starts from point (0,0), and then (0,1), (1,0), (0,2), (1,1), (2,0), etc., until the first point encountering the red tongue is found. The first point is called the left oblique point.

2. The searching order of the right oblique starts from the top right point (m, n), and then (m-1, n), (m, n-1), (m-2, n), etc., until the first point encountering the red tongue is found. The first point is called the right oblique point. The middle point between the left and right oblique points is defined as the root point of the tongue. It is shown as a star point in Figure 3(b) and is marked as symbol “A.”

#### Locating the center point

**Figure 4:**
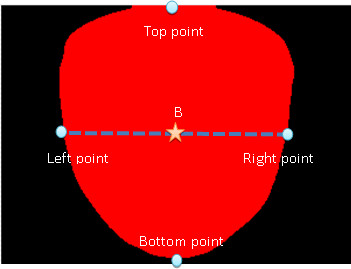
The star marks the center of the tongue image.

**Figure 5:**
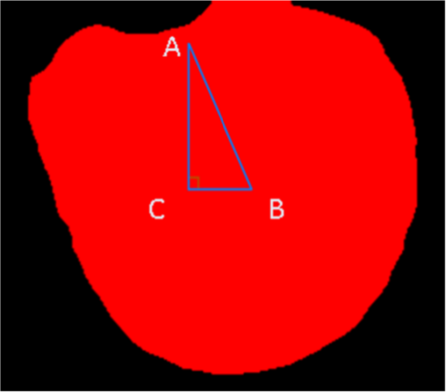
The tongue image of a stroke patient, and the related deviation angle.

The steps for finding the tongue center point are detailed as follows:

1. The vertical coordinate of the tongue center point: The top point and bottom point in Figure 4 are the highest and lowest points of the tongue, respectively. The value of the vertical coordinate of the tongue center point is determined by the average value of the vertical coordinate of the top and bottom points in the tongue, as shown in Figure 4.

2. The horizontal coordinate of the tongue center point: The left point and right point in Figure 4 are defined by the intersection points between the tongue edge and the horizontal line, which crosses the middle point between the top and bottom points. The horizontal position of the tongue center point was thus derived by the horizontal value of the middle point between the left and right points in the tongue, as shown in Figure 4. As soon as the horizontal position of the tongue center point was found, the center point of the tongue was specifically determined. It was shown as a star point in Figure 4 and marked as symbol “B.” The tongue center point means the center of the tongue; thus, it may deviate with an angle compared to the vertical line in stroke patients, as shown in Figure 5.

#### Evaluating the tongue deviation angle

The value of the vertical coordinate of point C in Figure 5 is equivalent to that of point B, and the value of the horizontal coordinate of point C is equivalent to that of point A. Thus, point C is defined, and ∟C in Figure 5 forms a right angle. ∟A in Figure 5 is the tongue deviation angle used to recognize stroke patients. ∟A is then calculated by the following:

(1) $$∟A=\tan^{-1}\frac{BC}{¯AC¯}\text{.}$$

### Reproducibility of the tongue deviation angle

Both operators were given a brief practical introduction to the technique of the tongue deviation angle, and then they performed 30 practice measurements over 5 days before embarking on the experiments. After taking the first capture of the tongue image by operator 1 and a 10-minute rest, we conducted the second capture by operator 2 in order to assess the reproducibility of the tongue deviation angles between operators. Next, after a 10-minute rest, we conducted the third capture again by operator 1 to assess the within-operator difference. There were 25 subjects involved in the reproducibility experiments. The mean and standard deviations of the capture on tongue deviation angles are listed in Table 1.

**Table 1:** Analysis of reproducibility of the tongue deviation angle

**Variable**	**Mean (degree)**	**Standard deviation (degree)**
1st measurement (operator 1)	1.63	0.14
2nd measurement (operator 2)	1.57	0.16
3rd measurement (operator 1)	1.48	0.13

SPSS software (SPSS 12.0, SPSS Inc., USA) was used to compute the Intra-class correlation coefficient (ICC). ICCs between the first and second measurement, and between the first and third measurement, are 0.85 and 0.89, respectively. The aforementioned results imply the excellent reliability for the intra-rater and inter-rater analyses. Consequently, there is high reproducibility in the measurements of the tongue deviation angle.

## Results

The subjects enrolled in the experiment included three groups. The first group comprised 25 stroke patients undergoing treatment, ranging between 55 and 82 years of age (15 males and 10 females). The second group comprised 25 TIA patients undergoing treatment, ranging between 53 and 79 years of age (16 males and 9 females), and the third group comprised 25 normal subjects with no stroke or TIA, ranging between 52 and 80 years of age (14 males and 11 females). The age distribution of the three groups had no statistical differences (*p* < 0.001). None of the subjects in the stroke group and the TIA group overlapped.

The members of the three groups had tongue images captured from 9/10/2011 to 3/05/2012. During this time, the subjects were visually inspected by two physicians (named “A” and “B”) using the same standard to identify whether the subjects had tongue deviations. The tongue was observed from the front with the examiner manually correcting any confounding mouth asymmetry. The position and configuration of the median raphe in relation to the nasal bridge were used to estimate the deviation. All participants were asked not to imbibe any alcoholic or caffeinated beverages on the day of the experiment. The experiment protocol was approved; written informed consent was obtained from all of the participants before they enrolled in this study.

**Figure 6:**
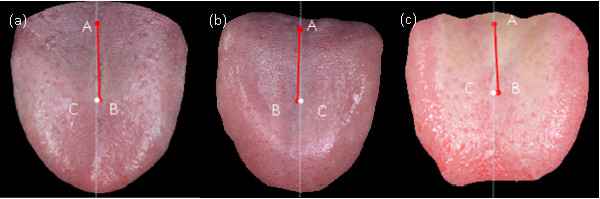
The tongue images of 3 normal subjects respectively with deviation angles = 0.74, 1.2, and 2.5 degrees.

**Figure 7:**
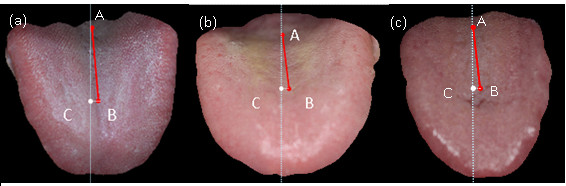
The tongue images of 3 TIA patients respectively with deviation angles = 4.3, 6.3, and 6.6 degrees.

**Figure 8:**
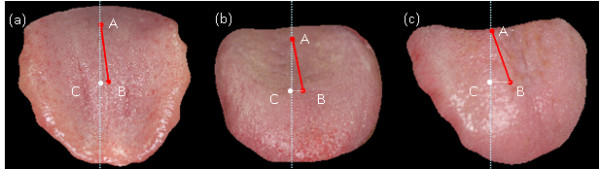
The tongue images of 3 stroke patients respectively with deviation angles = 8.1, 10.8, and 19.5 degrees.

Figure 6(a), 6(b) and 6(c) show the tongue images of 3 typical normal subjects, respectively with the tongue deviation angles of 0.74, 1.2, and 2.5 degrees. Figure 7(a), 7(b) and 7(c) show the tongue images of 3 typical TIA subjects, respectively with the tongue deviation angles of 4.3, 6.3, and 6.6 degrees. Figure 8(a), 8(b) and 8(c) show the tongue images of 3 typical stroke patients, respectively with the tongue deviation angles of 8.1, 10.8 and 19.5 degrees. Table 2 shows a summary of the means and standard deviations for the clinical characteristics of the three groups, including age, weight, height, BMI (Body Mass Index), and tongue deviation angle.

**Table 2:** Clinical characteristics of the stroke group, TIA group, and normal group

	**Stroke group**		**TIA group**		**Normal group**	
**Variable**	**Mean**	**Standard deviation**	**Mean**	**Standard deviation**	**Mean**	**Standard deviation**
Age (years)	67.5	10.3	66.7	9.1	66.5	9.7
Weight (kg)	69.5	11.8	66.7	10.6	65.6	10.1
Height (cm)	163.2	7.4	164.9	9.1	166.5	8.5
BMI (kg/m^2^)	26.5	2.3	25.9	2.0	24.3	1.9
Deviation Angle (degree)	6.9	3.1	4.9	2.0	1.4	0.8

**Figure 9:**
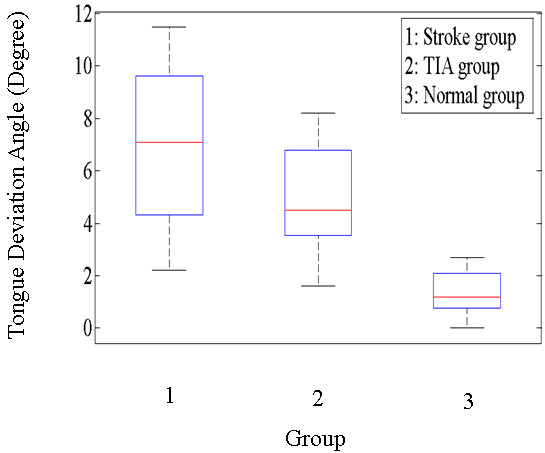
The box plot of the distribution of the stroke, TIA, and normal groups.

The means and standard deviations of the stroke, TIA, and normal groups were 6.9 ± 3.1, 4.9 ± 2.0 and 1.4 ± 0.8 degrees, respectively. Figure 9 shows the box plot of the distribution of the stroke, TIA, and normal groups. We used the Shapiro-Wilk test in SPSS software to verify the normality of the stroke, TIA, and normal groups. All the corresponding *p*-values are greater than 0.05; thus, the distributions of the stroke, TIA, and normal groups are normal, respectively. Next, analyzed by unpaired Student’s *t*-test, the *p-*value between the stroke and TIA groups was 0.015 (>0.01), indicating no significant difference in the tongue deviation angle. The *p-*values between the stroke and normal groups, as well as between the TIA and normal groups, were both less than 0.01. These results show the significant differences in the tongue deviation angle between the patient groups (stroke and TIA patients) and the normal group. These results also imply that the tongue deviation angle can effectively identify the patient groups (stroke and TIA patients) and the normal group. With respect to the visual examination of physician “A,” 40% of stroke patients, 24% of TIA patients, and 4% of normal subjects were found to have tongue deviations. As for the visual examination by physician “B,” 32% of stroke patients, 16% of TIA patients, and 0% of normal subjects were found to have tongue deviations [5,16]. 

**Figure 10:**
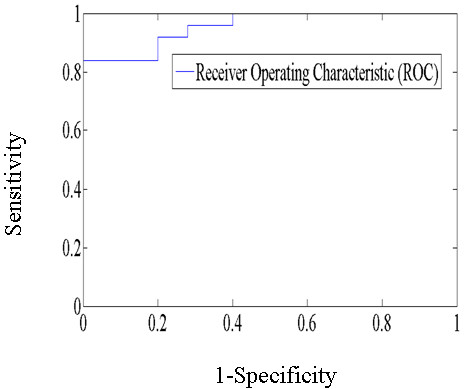
The receiver operating characteristic curve.

25 TIA patients and 25 normal subjects were included in the sensitivity analysis. We used the receiver operating characteristic curve (ROC) shown in Figure 10 to assess the sensitivity of the proposed method to evaluate the occurrence of a stroke. The Area Under Curve (AUC) (= 0.96) indicates good discrimination. The optimum operating point of the ROC curve corresponds to the threshold value of the tongue deviation angle of 3.2°. Thus, a tongue deviation angle greater than 3.2° may preliminarily predict a risk of a stroke.

## Discussion

Various techniques may assist in monitoring the brain. Examples include continuous transcranial Doppler, near-infrared spectroscopy, measurement of tissue oxygen, somatosensory evoked potentials and continuous electroencephalography (cEEG). For example, previous studies have shown that EEG provides physiological information following a stroke in the lesion site relating to the aspects of location, grade of damage, and physiological recovery [17-19]. Quantitative electroencephalography (qEEG) measures of delta (1-4 Hz) power and delta/alpha ratio (DAR) have been shown to be relevant to the site of ischemic lesion and effective in predicting the recovery from a stroke as well [17,20,21]. In addition, the brain symmetry index (BSI) was proposed to quantify the ischemic damage, which was also related to clinical acute ischemic hemispheric stroke [18,22]. Furthermore, the effectiveness of nonlinear analysis and detrended fluctuation analysis (DFA) was also reported in the case of subcortical strokes [17,23].

The tongue is a sensitive area of the human body as a result of its extensive neural controls. Consequently, even minor hypoglossal nerve damage resulting from a stroke may have a significant impact on the shape and motion of the tongue. Thus, the tongue deviation angle may be an indicator for determining the health or balance status of the nervous system before we resort to using expensive equipment. The experimental results indicated that almost all the participants, including stroke patients, TIA patients, and normal subjects had a nonzero deviation angle. The major differences existed in the degree of deviation, i.e., serious, moderate, or light deviation. Generally speaking, the stroke patients had larger average deviation angles (mean = 6.9 degrees), the TIA patients had smaller average deviation angles (mean = 4.9 degrees), and normal subjects had the smallest average deviation angles (mean = 1.4 degrees). Consequently, the clinical physician should not identify stroke patients based on the presence or absence of tongue deviation; instead, the physician should evaluate the degree of the tongue deviation angle.

With regard to the experimental results of visual inspection, there was a variation to some extent between physicians “A” and “B.” This is not surprising since visual illusion is a common occurrence for most people. A visual illusion is characterized by visually perceived images that differ from objective reality. The information gathered by the eye is processed in the brain to give a perception that does not conform to a physical measurement of the stimulus source [24-26]. For example, Figure 7(a), 7(b), 7(c), 8(a) and 8(b) present the images of 5 patients’ respective tongues with the deviation angles of 4.3, 6.3, 6.6, 8.1 and 10.8 degrees. We compare these with all the normal subjects, whose deviation angles show a distribution of 0 ~ 3 degrees. Even for such an obvious angle distinction in numeric values between the patients and the normal group, it is hard to determine by visual examination that the 5 patients have tongue deviations. Hence, these cases were diagnosed as “no tongue deviation” by both physicians “A” and “B.” The main reason for this may be that these angles are beyond the ability of the ordinary eye to distinguish from surrounding objects.

According to previous research, the percentage of stroke patients with tongue deviation is about 29% [5]. Due to visual illusion, the percentage is not high enough to make a case for allowing tongue deviation be a dependable indicator of stroke. Therefore, because relying on the eyes to determine the measurement of the deviation angle allows too much room for error, a quantification method is essential for clinical application. In this study, we developed a quantification method of tongue deviation angle and verified its effectiveness. We also found that even normal subjects have nonzero tongue deviation angles. The major differentiating factor between normal subjects and stroke patients is the degree of the tongue deviation angle. Using the tongue deviation angle of 3.2° discussed above as the threshold value, 84% of TIA patients and 88% of stroke patients are considered to have obvious tongue deviations. The percentages are much higher than that in reference 5, and they are high enough to be an indicator of stroke.

## Conclusion

A stroke is often referred to by doctors as a cerebrovascular accident, but a stroke is rarely an “accident.” The underlying conditions of a stroke are usually present for years before a stroke occurs, although the symptoms of a stroke may occur suddenly. The mean value of the tongue deviation angles of the TIA patients is less than that of the stroke patients, which explains the above statement. TIAs have the same symptoms as a stroke, but they are temporary and do not usually cause long-term brain damage. Just like full strokes, TIAs need emergency treatment and should not be ignored. A person who has had a TIA is at greater risk of having a stroke. Thus, a TIA, or mini-stroke, is a warning of an impending stroke. In other words, if we can identify the occurrence of TIA using the tongue deviation angle, we can present the warning sign of a stroke. By recognizing the warning sign and taking action, the patients may be able to prevent a stroke or reduce its severity because they are able to obtain medical help quickly.

Our results indicated that this quantification method can effectively distinguish between the patient groups (stroke and TIA patients) and the normal group. In particular, the method can distinguish between the TIA patients and the normal subjects. Because a TIA is a warning sign in the early stage of a stroke, this result demonstrated the feasibility of using the proposed quantification method as a prediction factor of a stroke. Capturing the tongue images of the patients at high risk of stroke, we may find the patients with larger tongue deviation angles in advance. In addition, if capturing the tongue image in a routine health examination, we may also identify the subjects with larger tongue deviation angles. Therefore, the presence of tongue deviation angles greater than 3.2° should alert the clinician to take the necessary precautions to avoid more dangerous complications for stroke patients. Preventing the complications of stroke will help to reduce a heavy burden on families and society. This quantification method of the tongue deviation angle is proposed for the first time for recognizing stroke patients. It improves the clinical tongue diagnosis of stroke. The experiments comparing normal and patient groups showed the method’s effectiveness. Due to the method’s simplicity, it may be applicable in future homecare or telehealth to present the early warning of the occurrence of a stroke or to show the prognosis.

## Competing interests

The authors declare that they have no competing interests.

## Authors’ contributions

CCW carried out the experiment design, analysis, and wrote the paper; SWH and SLH contributed to the collection and analysis of the tongue images; HCC, JSC and HL worked on the image processing of tongue segmentation and analysis. All authors read and approved the final manuscript.
